# Patterns of gray matter atrophy in atypical parkinsonism syndromes: a VBM meta-analysis

**DOI:** 10.1002/brb3.329

**Published:** 2015-03-11

**Authors:** Fang Yu, Daniel S Barron, Bundhit Tantiwongkosi, Peter Fox

**Affiliations:** 1 Department of Radiology, University of Texas Health Science CenterSan Antonio, Texas; 2 Research Imaging InstituteSan Antonio, Texas

**Keywords:** Neurodegenerative diseases, neuroimaging, Parkinson's disease

## Abstract

**Background and Purpose:**

Accurate diagnosis of Atypical Parkinsonian Syndromes (APS) is important due to differences in prognosis and management, but remains a challenge in the clinical setting. The purpose of our meta-analysis was to identify characteristic patterns of gray matter atrophy in Corticobasal Degeneration (CBD), Progressive Supranuclear Palsy (PSP), Multisystem-Atrophy Parkinsonian type (MSA-P), and Idiopathic Parkinson's Disease (IPD).

**Materials and Methods:**

Whole-brain meta-analysis was performed on 39 published voxel-based morphometry (VBM) articles (consisting of 404 IPD, 87 MSA-P, 165 CBD, and 176 PSP subjects) using the modified Anatomic Likelihood Estimation method. Based on these results, contrast analyses were then utilized to determine areas of atrophy shared by as well as unique to each disorder.

**Results:**

CBD was characterized by asymmetric gray matter atrophy in multiple cortical regions, while the thalamus-midbrain and insula were predominantly involved in PSP. The striatum and superior cerebellum were affected in MSA-P, while IPD demonstrated an anterior cerebral pattern. Although there was a mild overlap among PSP, CBD, and MSA-P, significant regions of atrophy unique to each disorder were identified, including (1) the superior parietal lobule in CBD (2) putamen in MSA-P (3) insula and medial dorsal nucleus in PSP.

**Conclusion:**

Our results suggest that there are characteristic patterns of atrophy in APS. Guided by these findings, future studies on the individual subject level may lead to the development of robust imaging biomarkers.

## Introduction

A multitude of movement disorders have been described in the neurological literature. Atypical Parkinsonism syndromes (APS) constitute a subset known to resemble Idiopathic Parkinson's Disease (IPD) on a clinical basis: multi-system atrophy (MSA), progressive supranuclear palsy, (PSP) and corticobasal degeneration (CBD) (Stamelou et al. 2013). Distinguishing these disorders from IPD, and from each other, is important given differences in prognosis and potential therapies, as well as the growing elderly population (Horwitz and Rowe 2011). Historically, this was based on physical exam findings of clinical Parkinsonism (e.g., rigidity, bradykinesia, tremors) with atypical features (e.g., autonomic symptoms, vertical gaze palsy). Unfortunately, this approach is limited because an individual's clinical presentation may be nonspecific until advanced disease.

Since the advent of clinical MRIs, there has been interest in establishing reliable imaging metrics as an alternative or supplement for this purpose (Jankovic et al. 2000). Quattrone et al. utilized manual measurements of brain stem parameters to distinguish PSP from MSA parkinsonian variant (MSA-P) and IPD with high sensitivity and specificity (Quattrone et al. 2008). Similar studies suggest that structural neuroimaging focusing on atrophy patterns may aid in the diagnosis of APS.

Voxel-based morphometry (VBM) is a technique that compares voxel-wise between-group differences in local brain morphology (Ashburner and Friston 2000). Many studies have used VBM to localize APS-related changes and reported regions of disease-related change. These individual VBM studies have limited generalizability and at times demonstrate inconsistent or even conflicting results, which is attributed to small sample sizes and differences in processing algorithms (Laird et al. 2005; Keller and Roberts 2008; Focke et al. 2011). The extensive VBM literature, however, permits a robust meta-analysis with a high level of statistical rigor. Such meta-analyses present a unique opportunity to localize consistent structural change across the many APS studies. This meta-analytic approach has been effectively utilized for various neurological diseases, including Huntington's disease (Lambrecq et al. 2012) and medial temporal lobe epilepsy (Barron et al. 2012). In medial temporal lobe epilepsy, the results of the VBM meta-analysis have informed numerous subsequent studies in individual patients (Barron et al. 2014, 2015).

The methodologies that form the basis of coordinate-based meta-analyses have undergone continued revisions. Among these, the latest iteration of the Anatomic Likelihood Estimations (ALE) algorithm improves upon the precision of its predecessors in identifying areas of structural change (Eickhoff et al. 2012). In the ALE technique, individual VBM foci are depicted as Gaussian probability distributions, which represent their underlying spatial uncertainty (Eickhoff et al. 2009, 2012). These distributions are pooled in a voxel-wise fashion within and across a group of experiments to generate a corresponding whole-brain ALE-map. Each voxel within the resulting ALE-map represents the probability of a specific experimental effect (i.e., gray matter volume loss). These maps are then tested against a null distribution, with a user-defined statistical threshold to determine clusters of significant meta-analytic convergence.

The purpose of our study was to employ the ALE meta-analytic algorithm to the spectrum of APS in order to systematically determine the most consistent patterns of gray matter atrophy. Conjunction and contrast analyses were then applied to identify significant regions of involvement both common as well as unique to each disorder. These results can guide future quantitative imaging analyses on the individual patient level.

## Materials and Methods

### Literature search and inclusion

Internet searches were performed of the PubMed Database for VBM studies of APS (e.g., for MSA, this would entail “Multi-system Atrophy” OR “MSA” AND “Voxel-based morphometry” OR “VBM”). The decision to focus on this particular subset of APS was based on an insufficient number of VBM studies for other disorders (fewer than four papers), such as multi-system atrophy-cerebellar type. From the studies returned, each was then individually reviewed to determine if it met the inclusion criteria (Table 1).

**Table 1:** Inclusion criteria and papers eliminated

Filter	Description	Rationale	Number of papers	Number eliminated
	CBD; MSA-P/C; PD; PSP		CBD; MSA-P; IPD; PSP	CBD; MSA-P; IPD; PSP
Internet Search of Pubmed	[(“Corticobasal Degeneration” OR “Corticobasal Degeneration Syndrome”); (“Multisystem-Atrophy” OR “MSA”); (“Parkinsons Disease” OR “PD” OR “IPD); (“PSP” OR “Progressive Supranuclear Palsy”)] AND (“voxel” OR “voxel-based morphometry” OR “VBM”)	Standard relevance criteria	34; 44; 339; 71	
Relevance	Published in English, peer-reviewed journals; reported x-y-z coordinates; whole-brain VBM in Talairach or MNI space	Diagnostic criteria needed for VBM meta-analysis	12; 10 30; 15	22; 34; 309; 56
Experimental Contrast	Compared to healthy controls; gray matter reductions; more than 5 subjects.	Allows for contrast analysis between disease groups	8; 7; 16; 12	4; 3; 14; 3
Nonredundancy	Only one paper per author/group.	Some groups analyzed the same set of patients across multiple papers.	8; 6; 16; 12	0; 1; 0; 0

VBM, voxel-based morphometry; CBD, corticobasal degeneration; MSA-P, multi-system atrophy Parkinsonian type; IPD, idiopathic Parkinson's disease; PSP, progressive supranuclear palsy; MNI, montreal neurological institute; VBM, voxel-based morphometry.

Our search yielded 39 APS papers (some papers evaluated more than one disorder), which are detailed in Table S1. Specifically, there were 16 papers, 110 coordinate foci, and 404 subjects for IPD, while MSA-P consisted of 6 papers, 85 foci, and 87 subjects. Additionally, 8 papers, 99 foci, and 165 subjects were found for CBD, while the PSP search yielded 12 papers, 122 foci, and 176 subjects. The diagnoses were largely established on clinical grounds. Specifically, MSA-P was diagnosed using the Consensus Criteria (Litvan et al. 2003), while the National Institute of Neurological Disorders and Stroke and the Society for PSP (NINDS-SPSP) criteria was used for PSP. Among the PSP studies, Ghosh et al. and Cordato et al. obtained postmortem neuropathologic confirmation in a portion of their subjects (9 of 22 and 5 of 21, respectively) (Cordato et al. 2005; Ghosh et al. 2012). Within the CBD group, only one study obtained pathological verification (Lee et al. 2011), while the remainder were based on findings by trained neurologists and published clinical criteria. Of the IPD group, 11 studies established their diagnoses based on the UK Parkinson's Disease Society Brain Bank criteria, with the remaining three using other published clinical criteria.

### Anatomic likelihood estimation

The VBM experiments for each set of movement disorders were retrieved, and the associated coordinates exported from the Brainmap database (www.brainmap.org) using Sleuth 2.3 (Laird et al. 2005). Experiments not available in the database were manually encoded using Scribe 2.3.1. Coordinates in Talairach & Tournoux (T&T) space were converted to Montreal Neurological Institute (MNI-152) space using the icbm2tal transform (Lancaster et al. 2007).

The modified anatomic likelihood estimation (ALE) algorithm was then utilized to determine spatial concordance among the VBM foci (Eickhoff et al. 2012). This approach to VBM meta-analyses seeks to determine the above-chance convergence between experiments, rather than between foci, and thus where in the brain convergence is higher across all included studies. In distinction to the fixed effects inference (between foci convergence), results derived from the random effects inference can be generalized to experiments outside of the analysis (Eickhoff et al. 2009).

Recent improvements to the ALE algorithm featured in GingerALE 2.3.1 include: (1) the implementation of limitations that an individual experiment can have across the group (2) the addition of cluster-level inference threshholding, which is better suited for topological features-derived statistical maps (3) replacing the highly time-consuming nonparametric empirical permutation algorithm for testing true convergence of foci with a nonlinear histogram integration Monte-Carlo-based approach.

For each set of disorders, ALE analyses were performed using a cluster-level threshold *P *< 0.05, a permutation threshold of 1000, and a false discovery rate (FDR) pN < 0.05 (unlike previous iterations, a cluster size volume threshold is not required for this algorithm). Mango software (http://ric.uthscsa.edu/mango) was then employed to visualize the ALE results, which were overlaid on the MNI-152 brain template in MNI coordinate space. A subset of papers listed their results as being in T&T coordinate space, without clarifying whether they referred to labels or coordinates. When possible, clarification was sought through electronic correspondence with the referring authors. For the remaining studies, separate ALE analyses for both MNI and T&T space were performed, and the one generating the highest level of convergence was selected. Leave-one-out sensitivity analyses were then performed by repeating the analysis with consecutive exclusion of each study to confirm robustness of the results.

### Contrast and conjunction analyses

Additional analyses were performed using the contrast analysis function in GingerAle to determine statistically significant differences in gray matter change between each individual disorder and the others. For the purposes of our study, the ALE image of each disorder was contrasted against an ALE image generated from a combination of the other three disorders. In order to correct for differences in study sizes, simulated data were generated from the original data sets, and randomly divided into partitions of equal size as the input data (Eickhoff et al. 2011).

For example, CBD contained 99 foci, while the remaining three disorders consisted of 317 foci (IPD, MSA-P, and PSP). The pooled APS data of 416 foci were therefore randomly divided into groups of 99 and 317 foci. ALE scores were then calculated from this simulated data and compared to the ALE scores of the two input data sets at each voxel in the brain. This was repeated for 10,000 iterations, yielding a null distribution for the difference in ALE scores between CBD and the other APS. The observed difference in ALE scores was then tested against this null hypothesis at each voxel, generating a voxel-wise *P*-value image that was thresholded with a FDR pN < 0.05 and a minimum cluster size of 200 mm^3^. The results were then overlaid on the MNI-152 brain template in Montreal Neurological Institute (MNI) coordinate space.

Separate conjunction analyses were also performed for each disorder to assess for common regions of convergence among the APS. This was derived from the voxel-wise minimum value of the input ALE images. The resulting conjunction image reflects the statistically significant similarities between the data set of interest (e.g., CBD), and the rest of the pooled disorders.

## Results

### ALE results

**Figure 1:**
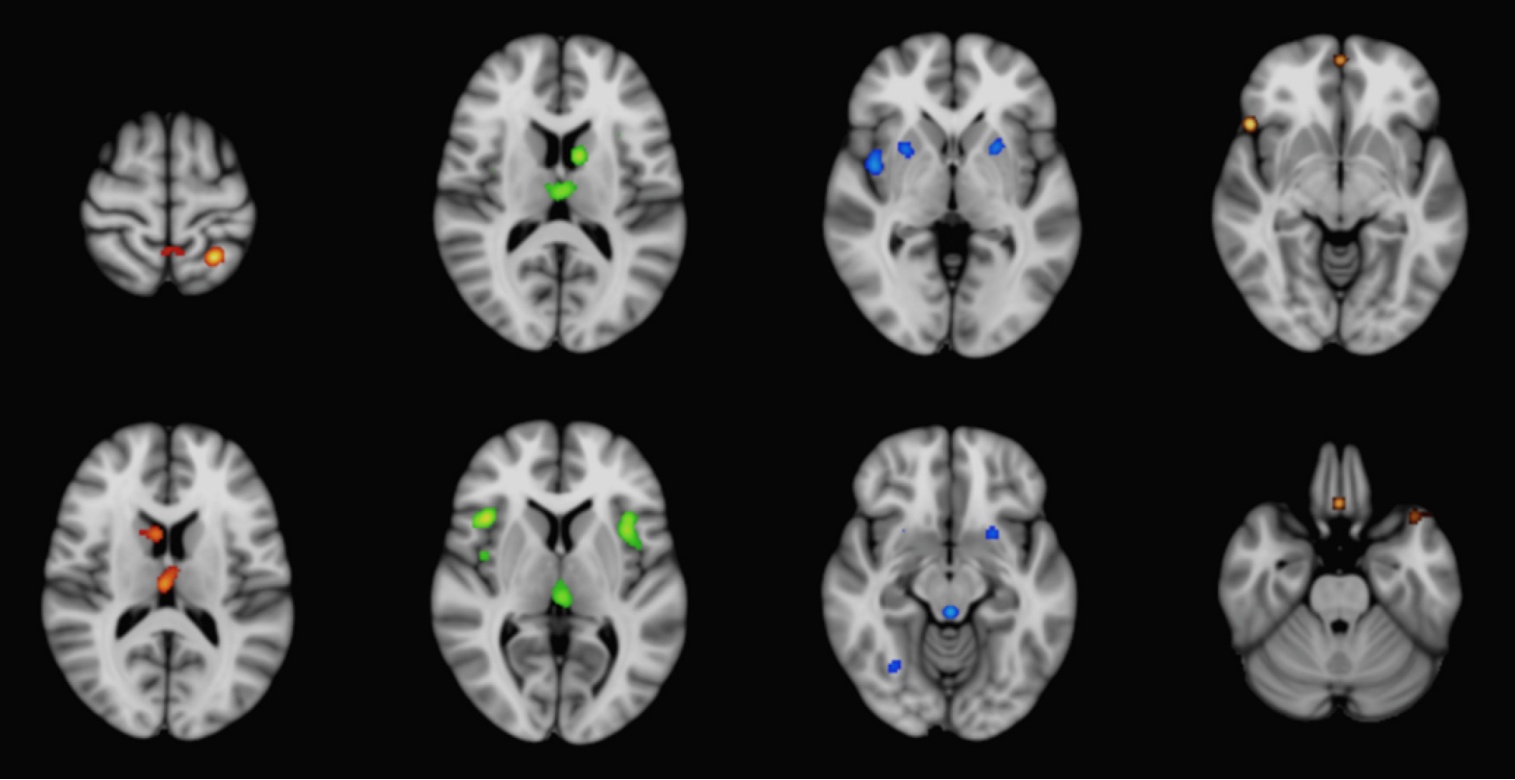
ALE meta-analysis results for significant gray matter atrophy in CBD (red), PSP (green), MSA-P (blue), and IPD (gold) compared to control subjects, superimposed on the MNI-152 template brain. ALE, anatomic likelihood estimation; CBD, corticobasal degeneration; PSP, progressive supranuclear Palsy; MSA-P, multisystem atrophy Parkinson-type; IPD, idiopathic Parkinson's disease; MNI, Montreal Neurological Institute.

The results from the ALE meta-analysis of gray matter reduction for the four disorders compared to controls are detailed in Table 2 and Figure 1. PSP demonstrated significant convergence for gray matter reduction centered in the medial thalamus, which was represented in 58% of studies. This cluster was also the largest among the APS, with extension to the midbrain. The bilateral insula were also involved, albeit to a lesser extent. Other significant clusters were found in the left caudate head and medial frontal gyrus.

**Table 2:** Anatomic likelihood estimations results

Disorder	Cluster number	Weighted center (*x*,*y*,*z*) in MNI space	Anatomic label	Volume (mm^3^)	Maximum ALE value (×10^−3^)	Total foci	Percent of studies represented	Contributors
CBD	1	1, −15, 16	Left Thalamus	1192	14.5	4	50%	Borroni B (2008), Pardini M, (2009), Huey ED, (2009), Lee SE (2011)
	2	−25, −51, 64	Left superior parietal lobe (BA 7)	1104	17.6	4	38%	Borroni B (2008), Boxer AL (2006), Morgan B (2011)
	3	9, 10, 14	Right caudate body	680	12.8	3	38%	Pardini M (2009), Huey ED (2009), Boxer AL (2006)
			Right caudate body		8.3			
	4	50, −26, −1	Right superior temporal gyrus (BA 22)	488	13.0	2	25%	Grossman M (2004), Morgan B (2011)
	5	−30, −6, 50	Left middle frontal gyrus (BA 6)	448	12.4	2	25%	Boxer AL (2006), Lee SE (2011)
	6	−1, −48, 66	Left parietal lobe precuneus (BA 7)	368	9.4	3	25%	Grossman M (2004), Huey ED (2009)
	7	−48, 17, 39	Left middle frontal gyrus (BA 8)	344	10.2	2	25%	Borroni B (2008), Grossman M (2004)
MSA-P	1	43, 1, −1	Right Claustrum	1184	12.8	4	67%	Tzarouchi LC (2010), Chang CC (2009), Minnerop M (2007), Brenneis C SK (2003)
	2	−23, 10, −5	Left Putamen	1120	12.3	4	50%	Tzarouchi LC (2010), Minnerop M (2007), Brenneis C S. K. (2003)
			Left Putamen		10.7			
	3	2, −31, −8	Right Thalamus	1072	15.8	4	50%	Tzarouchi LC (2010), Minnerop M (2007), [61]
	4	26, 9, −3	Right Putamen	664	11.9	3	50%	Tzarouchi LC (2010), Minnerop M (2007), Brenneis C S. K. (2003)
	5	31, −64, −13	Right Cerebellum (declive)	360	10.4	2	33%	Tzarouchi LC (2010), [61]
PSP	1	0, −21, 5	Left Thalamus	4064	18.2	15	58%	Padovani A (2006), Agosta F (2010), Cordato NJ (2005), Whitwell JL D. J. (2013), Lehéricy S (2010), Boxer AL (2006), Lagarde J (2013)
			Left Thalamus		15			
			Left Red Nucleus		14.8			
			Left Thalamus		14.7			
	2	−38, 12, 5	Left Claustrum	2096	16.3	8	50%	Brenneis C S. K. (2004), Takahashi R (2011), Padovani A (2006), Cordato NJ (2005), Whitwell JL D. J. (2013), Boxer AL (2006)
			Left Insula (BA 13)		15.6			
			Left insula (BA 13)		10.8			
	3	42, 19, 5	Right Insula (BA 13)	1336	19.2	5	33%	Ghosh BC (2012), Brenneis C S. K. (2004), Padovani A (2006), Boxer AL (2006)
	4	−10, 5, 13	Left caudate body	1048	17.8	4	33%	Agosta F (2010), Cordato NJ (2005), Whitwell JL D. J. (2013), Boxer AL (2006)
	5	41, −2, 6	Right Insula (BA 13)	496	11.3	3	25%	Ghosh BC (2012), Brenneis C S. K. (2004) Agosta F (2010)
IPD	1	50, 22, −6	Right inferior frontal gyrus (BA 47)	776	16.9	3	19%	Burton EJ (2004), O'Callaghan C (2013) Pereira JB (2009)
	2	1, 27, −27	Left medial frontal gyrus (BA 25)	448	13.6	2	13%	Nagano-Saito A (2005), O'Callaghan C (2013)
	3	1, 58, −7	Left medial frontal gyrus (BA 10)	448	14.9	3	13%	Dalaker TO (2010), Nishio Y (2010)
	4	−42, 20, −25	Left inferior frontal gyrus (BA 47)	440	11.9	3	19%	Meppelink AM (2011), Ramírez-Ruiz B (2007), Pereira JB (2009)
			Left superior temporal gyrus (BA 38)		9.0	2	13%	
	5	35, 36, 24	Right middle frontal gyrus (BA 9)	360	12.1	2	13%	Burton EJ (2004), Kostić VS (2010)
	6	−31, 31, −12	Left inferior frontal gyrus (BA 47)	304	11.4	2	13%	Dalaker TO (2010), Kostić VS (2010)
	7	−7, 55, 17	Left medial frontal gyrus (BA 9)	272	11.0	2	13%	Kostić VS (2010), Pereira JB (2009)

BA, Brodman's area; CBS, corticobasal syndrome; CBD, corticobasal degeneration; MSA-P, multisystem atrophy Parkisonian type; PCA, posterior cerebral atrophy; IPD, idiopathic Parkinson's Disease; PSP, progressive supranuclear palsy.

For CBD, the most consistent regions of gray matter reduction were located in the thalamus and left superior parietal lobule. These regions were represented in up to 50% of the studies from this group. Additional clusters of gray matter reduction included the right caudate, as well as several cortical regions, which were situated predominantly in the left cerebral hemisphere.

The bilateral putamen and right claustrum were the most significantly involved regions in MSA-P. Additional areas of significant convergence were present in the thalamus and right cerebellum (declive). In comparison, IPD demonstrated significant convergence in a predominantly anterior cortical distribution, with the largest cluster situated in the right inferior frontal gyrus. This was also the only IPD cluster to exceed 500 mm^3^ in size; none were represented in more than 20% of the studies.

### Contrast analysis results

**Figure 2:**
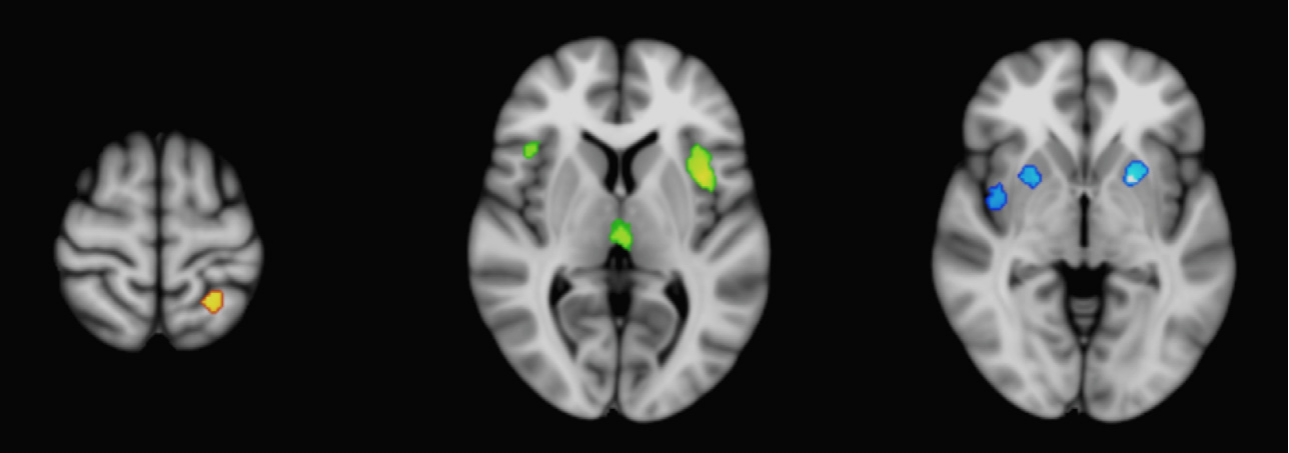
Contrast analysis results for significant gray matter atrophy unique to CBD (red), PSP (green), and MSA-P (blue), superimposed on the MNI-152 brain template. CBD, corticobasal degeneration; PSP, progressive supranuclear Palsy; MSA-P, multisystem atrophy Parkinson-type.

Contrast analyses were performed to determine if there were regions of significant gray matter volume loss that could distinguish each individual disorder from the others (Fig.2). CBD was characterized by atrophy of the left superior parietal lobe, whereas PSP was notable for involvement of the medial dorsal nucleus of the thalamus and bilateral insula. Meanwhile, MSA-P was distinguished by involvement of the bilateral putamen and right claustrum. IPD did not demonstrate significant distinguishing regions.

### Conjunction analysis results

**Figure 3:**
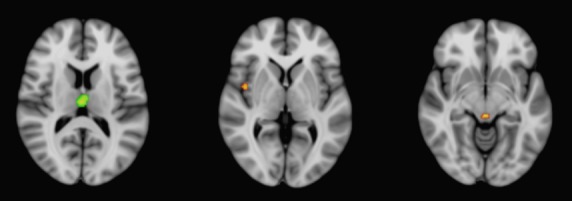
Conjunction analysis results for shared regions of gray matter atrophy, superimposed on the MNI-152 brain template. CBD and PSP shared involvement of the superior thalamus, while PSP and MSA-P involved the right insula and midbrain (red). CBD, corticobasal degeneration; PSP, progressive supranuclear Palsy; MSA-P, multisystem atrophy Parkinson-type; MNI, Montreal Neurological Institute.

Conjunction analyses found the superomedial thalamus to be a region of gray matter atrophy common to both CBD and PSP (Fig.3). Meanwhile, the right insula (Brodmann Area 13) as well as midbrain were involved in PSP and MSA-P. IPD did not demonstrate significant overlap in gray matter atrophy with other APS.

## Discussion

Our VBM meta-analysis identified distinctive patterns of gray matter reduction in CBD, PSP, MSA-P, and IPD. While mild overlap in gray matter atrophy existed between CBD and PSP, as well as PSP and MSA-P, there were regions of atrophy distinctive to each disease, including the left parietal lobe in CBD, thalamus and insula in PSP, and putamen in MSA-P.

PSP is a neurodegenerative tauopathy that manifests with vertical gaze palsies, postural instability, bradykinesia, and dementia (Boxer et al. 2006). On the other hand, CBD, which is also a tauopathy, classically presents with asymmetric motor and cortical sensory dysfunction (Rankin et al. 2011; Rohrer et al. 2011). Our results indicate that a distinction between these diseases may be made on a neuroanatomical basis. Compared to PSP, CBD demonstrated a more asymmetric, posterior cortical pattern of gray matter atrophy (Massey et al. 2012). Neuropathologic studies corroborate these findings, revealing more widespread supratentorial and cortical involvement in CBD (Forman et al. 2002; Schofield et al. 2005). These morphological patterns may account for the observed differences in clinical presentation.

Varying degrees of Parkinsonian features in tandem with cerebellar and autonomic dysfunction distinguish MSA as a clinical syndrome (Wenning et al. 1997; Gilman et al. 2008). Unlike the tauopathies, alpha-synuclein glial inclusions represent the primary histopathology findings (Ozawa et al. 2007; Petrovic et al. 2012). Our results indicate that gray matter atrophy was concentrated in the putamen and claustrum (Kraft et al. 1999, 2002; Yekhlef et al. 2003). Consistent with postmortem pathologic studies, the pulvinar, midbrain, and superior cerebellum are also involved, likely contributing to its unique symptomatology (Burn and Jaros 2001; Ramirez and Vonsattel 2014). In comparison, IPD was associated with a frontal cortical-predominant distribution of gray matter decrease, without significant involvement of the deep gray nuclei. Notably, these features did not persist on contrast analyses. The average age and disease duration were similar among the APS groups, which suggests that this observation is unlikely the result of earlier stage disease. Instead, gray matter reduction in IPD may be less pronounced than in other APS (Paviour et al. 2006; Berg et al. 2011; Massey et al. 2012).

There was mild overlap in gray matter atrophy among the different groups as indicated by the conjunction analyses. These findings may be responsible for the overlapping clinical features seen in early disease (Kimber et al. 2000). Although the underlying mechanism is unclear, it is possible that certain brain regions may have greater intrinsic susceptibility to multiple pathologies. For instance, the shared regions of involvement we identified (thalamus, insula, and midbrain) are known to demonstrate functional and structural connectivity with multiple brain regions, effectively acting as neural hubs (Cole et al. 2010; Crossley et al. 2014). Diseases that affect the underlying brain architecture would also be more likely to involve these regions. Additionally, it has been suggested that hubs may have higher metabolic demands, and are thus more prone to insults such as oxidative stress.

Taken together, our results indicate that there are distinctive patterns of gray matter involvement among the APS. However, cerebral atrophy is likely a later disease manifestation, which may limit its potential utility in early detection (Horwitz and Rowe 2011). Instead, greater value may be derived from implementation in disease staging, as well as elucidating the underlying neurophysiological basis. For example, recent diffusion tensor (DTI) and diffusion-weighted imaging (DWI) studies have shown more widespread supratentorial white matter involvement in CBD/CBS, and of the cerebellum in MSA, which correlate with our meta-analysis results (Borroni et al. 2008; Rizzo et al. 2008; Erbetta et al. 2009; Wang et al. 2011; Nicoletti et al. 2013). These findings also emphasize the relationship between white and gray matter involvement in APS.

There has been growing interest in characterizing neurological disorders using network-driven paradigms. This is particularly relevant to the APS, which demonstrate selective vulnerability of different brain regions (Horwitz and Rowe 2011). Efforts to characterize these networks utilizing functional connectivity have been carried out recently using resting-state fMRI (rsfMRI). Correlation with atrophy patterns may help to illuminate and validate the relevant functional components (Atluri et al. 2013). For instance, patterns of atrophy in several neurodegenerative disorders have been shown to correspond to known resting-state networks (Seeley et al. 2009; Zhou et al. 2010; Crossley et al. 2014). Whitwell et al. found reduced functional connectivity in PSP between the thalamus, prefrontal cortex, and striatum, which reflect the regions of involvement identified in our study (Whitwell et al. 2011). Although there is evidence that synaptic loss and axonal deterioration precede neuronal death in many neurodegenerative disorders, the sequence of connectivity derangements and atrophy over the course of disease remains unclear, especially at the early and preclinical stages (Paviour et al. 2006). Focusing on the brain regions highlighted in our meta-analysis, future longitudinal studies matching volumetric with connectivity data may help to elucidate this. Additional work is also required to establish the applicability of these techniques on the individual subject level, which may lead to the development of new robust biomarkers.

### Limitations

A potential limitation of our meta-analysis concerns disease diagnosis: most studies utilized published clinical criteria without histological correlation. Although clinical diagnoses for these disorders suffer from lower sensitivity, their specificities remain high (Litvan et al. 1997). In particular, the NINDS-SPSP criteria for PSP has been shown to have over 95% sensitivity and specificity in diagnosing subjects with the classic Richardson-type presentation (Litvan et al. 2003). Therefore, the subjects included in the studies were likely to have the ascribed diagnoses, with some variation between disorders. This is supported by the high degree of agreement between the VBM studies with neuropathologic confirmation and those based on clinical criteria alone. Unfortunately, individuals with early or atypical presentations (i.e., cognitive-predominant deficits) may have been overlooked, highlighting an inherent limitation of existing clinical criteria.

Another limitation of our study is that we were unable to rigorously assess the effects of disease duration and other clinical parameters on brain atrophy due to the limited VBM source data. Future studies aimed at addressing such questions will benefit from the results of our meta-analysis, specific to each APS subtype. Finally, although significant statistical rigor was exercised in utilizing the revised ALE meta-analytic technique, our study was nevertheless based on summarized stereotactic coordinates rather than raw imaging data. Thus, heterogeneity in VBM techniques of the individual studies may potentially influence our results, especially in the case of smaller data sets. In particular, segmentation of the brainstem gray matter nuclei is an area of technical weakness in VBM, which may account for the lack of significant involvement identified in this region (Focke et al. 2011; Shigemoto et al. 2013).

## Conclusion

Through the application of revised VBM meta-analytic techniques to APS, we demonstrated distinctive patterns of gray matter atrophy. In addition, through contrast analyses, significant patterns of involvement unique to each disorder were identified, including the superior parietal lobule in CBD, the putamen and claustrum in MSA, and the insula as well as medial dorsal nucleus in PSP. Future longitudinal studies focusing on these brain regions at the individual patient level, in conjunction with other advanced imaging techniques, may yield important biomarkers.

## Conflict of Interest

The author(s) have no grants, conflicts of interest, or disclosures to divulge that are applicable to this investigation.

## Supporting Information

**Table S1** Overview of included VBM studies.
