# Supplementary material for: Patterns of gray matter atrophy in atypical parkinsonism syndromes: a VBM meta-analysis
Source: Brain Behav. 2015 Mar 11;5(6):e00329. doi: 10.1002/brb3.329 (PMC4467770; doi:10.1002/brb3.329)
Supplement: Supplementary file 1 [file brb30005-e00329-sd1.docx]

Supplemental Table 1: Overview of included VBM studies

| **Reference** | **Subjects** | **Number of subjects (females)** | **Age in years (SD)** | **Disease Duration in years (SD)** | **Template; Software** | **Statistical significance** | **Diagnostic criteria** |
| --- | --- | --- | --- | --- | --- | --- | --- |
|  | **CBD** |  |  |  |  |  |  |
| (Lee SE 2011) | CBD-CBS | 14 (10) | 66 (NA) | 6.7 (NA) | MNI; SPM5 | P < 0.001 (uncorrected) | Pathologic confirmation |
|  | HC | 44 (22) | 69 (5) |  |  |  |  |
| (Gross RG 2010) | CBS | 20 (11) | 67.4 (3.9) | 3.9 (2.0) | MNI; SPM5 | P < 0.05 (FDR) | Neurologist |
|  | HC | 8 (6) | 69.4 (9.8) |  |  |  |  |
| (Huey ED 2009) | CBS | 48 (23) | 66 (9) | 5 (NA) | MNI; SPM5 | P < 0.05 (FWE) | Clinical criteria (Boeve 2005); Neurologist & Neuropsychiatrist; |
|  | HC | 14 (6) | 60 (6) |  |  |  |  |
| (Pardini M 2009) | CBS | 25 (13) | 62 (9.0) | 4 (1.8) | MNI; SPM5 | P < 0.05 (FDR) | Neurologist & Neuropsychologist |
|  | HC | 12 (NA) | Matched |  |  |  |  |
| (Borroni B 2008) | CBDS | 20 (7) | 62.7 (8.0) | 2.0 (1.4) | MNI*; SPM2 | P < 0.05 (FDR) | Clinical criteria (Lang 1994); Neurologist & Neuropsychologist; |
|  | HC | 21 (13) | 65.6 (4.1) |  |  |  |  |
| (Boxer AL 2006) | CBDS | 14 (10) | 64.5 (5.9) | 5.6 (1.7) | MNI; SPM2 | P < 0.05 (FWE) | Neurologist |
|  | HC | 80 (43) | 67.9 (8.6) |  |  |  |  |
| (Grossman M 2004) | CBD | 9 (NA) | 64 (7) | 3.4 (1.5) | T&T; SPM99 | P < 0.001 (uncorrected) | Clinical criteria (Riley DE 2000) |
|  | HC | 12 (NA) | 68.5 (9.4) |  |  |  |  |
| (Morgan B 2011) | CBS + PCA | 10 CBS (NA); 3 PCA (NA) | 65.4 (8.4) | NA | MNI; SPM5 | P< 0.001 (uncorrected) | Clinical criteria (Riley DE 2000) |
|  | HC | 12 (NA) |  |  |  |  |  |
| **Total CBD patients** |  | **165** |  |  |  |  |  |
|  |  |  |  |  |  |  |  |
|  | **MSA-P** |  |  |  |  |  |  |
| (Shigemoto Y 2013) | MSA-P | 21 (13) | 62.9 (7.7) | 4.1 (2.2) | T&T; SPM8 | P < 0.05 (FDR) | Consensus Statement Criteria (W. G. Gilman S 2008) |
|  | HC | 30 (20) | 62.9 (7.7) |  |  |  |  |
| (Tzarouchi LC 2010) | MSA-P | 11 (2) | 61.9 (11.7) | 5.4 (2.5) | MNI; SPM5 | P < 0.001 (uncorrected) | Consensus Statement Criteria (L. P.-S. Gilman S 1999) |
|  | HC | 11 (3) | 64.6 (10.4) |  |  |  |  |
| (Chang CC 2009) | MSA-P | 13 (4) | 59.8 (8.1) | 2.6 (NA) | MNI; SPM2 | P < 0.05 (FDR) | Consensus Statement Criteria (L. P.-S. Gilman S 1999) |
|  | HC | 37 (17) | 55.5 (8.6) |  |  |  |  |
| (Tir M 2009) | MSA-P | 14 (9) | 63.6 (9.7) | 5.1 (2.2) | MNI; SPM2 | P < 0.05 (corrected) | Clinical (Litvan I and Committee 2003) |
|  | HC | 14 (9) | 59.2 (7.6) |  |  |  |  |
| (Minnerop M 2007) | MSA-P | 16 (8) | 62.8 (5.6) | 4.4 (2.3) | T&T; SPM2 | P < 0.01 (FDR) | Consensus Statement Criteria (L. P.-S. Gilman S 1999) |
|  | HC | 46 (24) | 58.7 (6.1) |  |  |  |  |
| (S. K. Brenneis C 2003) | MSA-P | 12 (NA) | 62 (6.6) | 2.8 (1.1) | MNI*; SPM99 | P < 0.05 (corrected) | Consensus Statement Criteria (L. P.-S. Gilman S 1999) |
|  | HC | 12 (NA) | Matched |  |  |  |  |
| **Total MSA-P patients** |  | **87** |  |  |  |  |  |
|  | **PSP** |  |  |  |  |  |  |
| (Lagarde J 2013) | PSP | 19 (12) | 65.9 (6.5) | 4.5 (1.8) | MNI; SPM8 | P < 0.001 (uncorrected) | NINDS-SPSP |
|  | HC | 18 (11) | 67.8 (5.2) |  |  |  |  |
| (Giordano A 2013) | PSP | 15 (7) | 68.9 (1.2) | 3.2 (1.3) | T&T; SPM8 | P< 0.05 (FWE) | NINDS-SPSP |
|  | HC | 15 (7) | 65.5 (6.1) |  |  |  |  |
| (D. J. Whitwell JL 2013) | PSP | 16 (8) | 71.8 (9.2) | 4.0 (2.3) | MNI; SPM5 | P < 0.05 (FDR) | NINDS-SPSP |
|  | HC | 20 (16) | 73.9 (6.3) |  |  |  |  |
| (Ghosh BC 2012) | PSP | 22 (7) | 71.1 (8.6) | 2.5 (NA) | MNI; SPM5 | P < 0.001 (uncorrected) | Clinical (Litvan I and Committee 2003); 9 subjects with pathologic confirmation |
|  | HC | 23 (9) | 71.4 (7.6) |  |  |  |  |
| (Takahashi R 2011) | PSP | 16 (5) | 64.9 (6.4) | 3 (NA) | MNI; SPM8 | P < 0.001 (uncorrected) | NINDS-SPSP |
|  | HC | 20 (8) | 64.8 (6.4) |  |  |  |  |
| (Agosta F 2010) | PSP-rc | 10 (3) | 62.5 (NA) | 3.8 (2.5-7) | MNI*; SPM5 | P < 0.05 (FWE) | NINDS-SPSP |
|  | PSP-P | 10 (3) | 67.3 (NA) | 5.1 (NA) |  |  |  |
|  | HC | 24 (11) | 63.8 (NA) |  |  |  |  |
| (Lehéricy S 2010) | PSP | 10 (4) | 66.9 (6.4) | 4.3 (1.0) | MNI; SPM5 | P < 0.05 (FDR) | NINDS-SPSP |
|  | HC | 9 (4) | 66.5 (4.8) |  |  |  |  |
| (Boxer AL 2006) | PSP | 15 (6) | 70.9 (6.9) | 4.8 (1.7) | MNI; SPM2 | P < 0.05 (FWE) | NINDS-SPSP |
|  | HC | 80 (43) | 67.9 (8.6) |  |  |  |  |
| (Padovani A 2006) | PSP | 14 (7) | 73 (5.6) | 3.1 (1.0) | MNI; SPM2 | P < 0.005 (FDR) | NINDS-SPSP |
|  | HC | 14 (7) | 65.6 (4.1) |  |  |  |  |
| (Cordato NJ 2005) | PSP | 21 (7) | 70.3 (6.4) | 3.9 (2.8) | MNI; SPM99 | P < 0.05 (corrected) | NINDS-SPSP; 5 subjects with pathologic confirmation |
|  | HC | 23 (9) | 71.6 (7.2) |  |  |  |  |
| (Price S 2004) | PSP | 12(5) | 65.3 (5.8) | 4.8 (1.7) | MNI*; SPM99 | P <0.05 (corrected) | NINDS-SPSP |
|  | HC | 12 (4) | 67.4 (4.6) |  |  |  |  |
| (S. K. Brenneis C 2004) | PSP | 12 (NA) | 67.5 (6.6) | 2.7 (0.9) | MNI*; SPM99 | P < 0.05 (corrected) | NINDS-SPSP |
|  | HC | 12 (matched) | 60 (5.8) |  |  |  |  |
| **Total PSP patients** |  | **176** |  |  |  |  |  |
|  |  |  |  |  |  |  |  |
|  | **IPD** |  |  |  |  |  |  |
| (Meppelink AM 2011) | IPD | 13 (NA) | NA | 7.9 (2.4) | MNI; SPM5 | P < 0.05 (corrected) | UK PD Society Brain Bank |
|  | HC | 14 (NA) |  |  |  |  |  |
| (O'Callaghan C 2013) | IPD | 25 (9) | 64.5 (7.3) | 7.3 (4.9) | MNI; FSL-VBM | P < 0.05 (FWE) | UK PD Society Brain Bank |
|  | HC | 15 (3) | 64.2 (4.9) |  |  |  |  |
| (Ibarretxe-Bilbao N 2012) | IPD | 16 (4) | 55.9 (8.1) | 3.0 (1.5) | MNI; SPM8 | P < 0.05 (FWE) | UK PD Society Brain Bank |
|  | HC | 15 (3) | 57.7 (9.5) |  |  |  |  |
| (Fernández-Seara MA 2012) | IPD | 25 (7) | 63.2 (6.6) | 5.6 (3.4) | MNI; SPM8 | P < 0.05 (cluster-corrected) | UK PD Society Brain Bank; Neurologist |
|  | HC | 34 (12) | 63.5 (6.6) |  |  |  |  |
| (Focke NK 2011) | IPD | 21 (6) | 65.2 (8.0) | 5.5 (3.4) | MNI; SPM8 | P < 0.05 (FWE) | UK PD Society Brain Bank |
|  | HC | 22 (9) | 69.3 (9.1) |  |  |  |  |
| (Kostić VS 2010) | IPD | 24 (11) | 65 (NA) | 5 (NA) | MNI; SPM 5 | P < 0.05  (FWE) | UK PD Society Brain Bank |
|  | HC | 26 (12) | 63 |  |  |  |  |
| (Nishio Y 2010) | IPD-NC | 27(14) | 65.6 (5.2) | 3.7 (2.9) | MNI; SPM5 | P < 0.001 (uncorrected) | UK PD Society Brain Bank; Neurologist |
|  | IPD-CI | 13 (1) | 67.6 (5.5) | 6.1 (5.8) |  |  |  |
|  | HC | 13 (6) | 63 (4.6) |  |  |  |  |
| (Dalaker TO 2010) | IPD | 42 (17) | 64.6 (9.6) | 2.8 (4.0) | MNI**; SPM5 | P < 0.05 (FDR) | Clinical (Gelb DJ 1999) |
|  | HC | 37 (15) | 63.9 (9.5) |  |  |  |  |
| (Camicioli R 2009) | IPD | 43 (19) | 70.7 (4) | 8.3 (4.5) | MNI; SPM2 | P < 0.01 (FDR) | (Camicioli R 2009) |
|  | HC | 43 (19) | 71 (4.5) |  |  |  |  |
| (Pereira JB 2009) | IPD | 36 (22) | 73.2 (5.6) | 11.8 (4.9) | MNI; SPM5 | P < 0.05 (FDR) | UK PD Society Brain Bank |
|  | HC | 20 (10) | 72.7 (6.7) |  |  |  |  |
| (Tir M 2009) | IPD | 19 (8) | 61.6 (7.6) | 6.6 (2.5) | MNI; SPM2 | P < 0.05 (corrected) | Clinical (Litvan I and Committee 2003) |
|  | HC | 14 (9) | 59.2 (7.6) |  |  |  |  |
| (Ramírez-Ruiz B 2007) | IPD | 20 (12) | NA | 10.6 (4.3) | T&T; SPM2 | P < 0.05 | UK PD Society Brain Bank |
|  | HC | 21 (12) | Matched |  |  |  |  |
| (Cordato NJ 2005) | IPD | 17 (4) | 67.7 (6.7) | 7.6 (2.9) | MNI; SPM99 | P < 0.05 (corrected) | Clinical (Gelb DJ 1999) |
|  | HC | 23 (9) | 71.6 (7.2) |  |  |  |  |
| (Summerfield C 2005) | IPD | 13 (NA) | 72.8 (4.9) | 10.6 (7.4) | MNI*; SPM99 | P < 0.001 (Uncorrected) | UK PD Society Brain Bank |
|  | HC | 13 (NA) | 70.1 (7.2) |  |  |  |  |
| (Nagano-Saito A 2005) | IPD | 19 (NA) | 62.6 (7.9) | 4.9 (4.3) | MNI; SPM2 | P < 0.05 (corrected) | Clinical (Calne DB 1992) |
|  | HC | 31 (NA) | 63.5 (8.8) |  |  |  |  |
| (Burton EJ 2004) | IPD | 31 (8) | 75.2 (5.2) | 3.6 (0.9) | T&T; SPM99 | P < 0.001 (uncorrected) | UK PD Society Brain Bank |
|  | HC | 36 (16) | 75.1 (6.6) |  |  |  |  |
| **Total IPD patients** |  | **404** |  |  |  |  |  |
|  |  |  |  |  |  |  |  |
|  |  |  |  |  |  |  |  |
|  |  |  |  |  |  |  |  |

CBS = Corticobasal Syndrome, CBDS = Corticobasal Degeneration Syndrome, CBD = Corticobasal Degeneration, FWE = Family Wise Error, FDR = False Discovery Rate, HC = Healthy Controls, MSA-C/P = Multisystem Atrophy Cerebellar/Parkisonian Type, NINDS-SPSP = National Institute of Neurological Disorders and Stroke and Society of Progressive Supranuclear Palsy, PCA = Posterior cerebral atrophy, IPD = Idiopathic Parkinson’s Disease, PD-NC = Parkinson’s disease without cognitive impairment, PD-CI = Parkinson’s Disease with Cognitive Impairment, PSP = Progressive Supranuclear Palsy, PSP-rc = PSP Richardson type, PSP-P = PSP Parkinsonian type, T&T = Talairach & Tournoux

* Refers to paper indicating “Talairach” in their results, without referencing a conversion or explicitly stating a coordinate system. These were handled as T&T labels and the coordinates themselves were in MNI space.

** T&T coordinates converted to MNI coordinate space using the Lancaster transform

# Bibliography

1. Agosta F, K. V. (2010). The in vivo distribution of brain tissue loss in Richardson's syndrome and PSP-parkinsonism: a VBM-DARTEL study. *Eur J Neurosci.* *, 32* (4), 640-7.
2. Borroni B, G. V. (2008). White matter changes in corticobasal degeneration syndrome and correlation with limb apraxia. *Arch Neurol.* *, 65* (6), 796-801.
3. Boxer AL, G. M.-T. (2006). Patterns of brain atrophy that differentiate corticobasal degeneration syndrome from progressive supranuclear palsy. *Arch Neurol.* *, 63* (1), 81-6.
4. Brenneis C, B. S. (2006). Cortical atrophy in the cerebellar variant of multiple system atrophy: A voxel-based morphometry study. *Mov Disord.* *, 21* (2), 159-65.
5. Brenneis C, S. K. (2004). Voxel based morphometry reveals a distinct pattern of frontal atrophy in progressive supranuclear palsy. *J Neurol Neurosurg Psychiatry* *, 75* (2), 246-9.
6. Brenneis C, S. K. (2003). Voxel-based morphometry detects cortical atrophy in the Parkinson variant of multiple system atrophy. *Mov Disord.* *, 18* (10), 1132-8.
7. Burton EJ, M. I. (2004). Cerebral atrophy in Parkinson's disease with and without dementia: a comparison with Alzheimer's disease, dementia with Lewy bodies and controls. *Brain* *, 127*, 791-800.
8. Camicioli R, G. M. (2009). Voxel-based morphometry reveals extra-nigral atrophy patterns associated with dopamine refractory cognitive and motor impairment in parkinsonism. *Parkinsonism Relat Disord.* *, 15* (3), 187-95.
9. Chang CC, C. Y. (2009). Cognitive deficits in multiple system atrophy correlate with frontal atrophy and disease duration. *Eur J Neurol.* *, 16* (10), 1144-50.
10. Cordato NJ, D. A. (2005). Clinical deficits correlate with regional cerebral atrophy in progressive supranuclear palsy. *Brain* *, 128*, 1259-66.
11. Dalaker TO, Z. R. (2010). Gray matter correlations of cognition in incident Parkinson's disease. *Mov Disord.* *, 25* (5), 629-33.
12. Focke NK, H. G. (2011). Individual voxel-based subtype prediction can differentiate progressive supranuclear palsy from idiopathic Parkinson syndrome and healthy controls. *Hum Brain Mapp.* *, 32* (11), 1905-15.
13. Ghosh BC, C. A.-C. (2012). Social cognitive deficits and their neural correlates in progressive supranuclear palsy. *Brain.* *, 135*, 2089-102.
14. Giordano A, T. A. (2013). Clinical and cognitive correlations of regional gray matter atrophy in progressive supranuclear palsy. *Parkinsonism Relat Disord.* *, 19* (6), 590-4.
15. Gross RG, A. S. (2010). Impaired information integration contributes to communication difficulty in corticobasal syndrome. *Cogn Behav Neurol.* *, 23* (1), 1-7.
16. Grossman M, M. C. (2004). What's in a name: voxel-based morphometric analyses of MRI and naming difficulty in Alzheimer's disease, frontotemporal dementia and corticobasal degeneration. *Brain* *, 127*, 628-49.
17. Huey ED, P. M. (2009). Association of ideomotor apraxia with frontal gray matter volume loss in corticobasal syndrome. *Arch Neurol.* *, 66* (10), 1274-80.
18. Lee SE, R. G.-T. (2011). Clinicopathological correlations in corticobasal degeneration. *Ann Neurol.* *, 70* (2), 327-40.
19. Lehéricy S, H. A. (2010). Magnetic resonance imaging lesion pattern in Guadeloupean parkinsonism is distinct from progressive supranuclear palsy. *Brain* *, 133*, 2410-25.
20. Meppelink AM, d. J. (2011). Regional cortical grey matter loss in Parkinson's disease without dementia is independent from visual hallucinations. *Mov Disord.* *, 26* (1), 142-7.
21. Minnerop M, S. K. (2007). Voxel-based morphometry and voxel-based relaxometry in multiple system atrophy-a comparison between clinical subtypes and correlations with clinical parameters. *Neuroimage* *, 36* (4), 1086-95.
22. Morgan B, G. R. (2011). Some is not enough: quantifier comprehension in corticobasal syndrome and behavioral variant frontotemporal dementia. *Neuropsychologia.* *, 49* (13), 3532-41.
23. Nagano-Saito A, W. Y. (2005). Cerebral atrophy and its relation to cognitive impairment in Parkinson disease. *Neurology* *, 64* (2), 224-9.
24. Nishio Y, H. K. (2010). Corticolimbic gray matter loss in Parkinson's disease without dementia. *Eur J Neurol. , 17* (8), 1090-7.
25. O'Callaghan C, N. S. (2013). Fronto-striatal atrophy correlates of inhibitory dysfunction in Parkinson's disease versus behavioural variant frontotemporal dementia. *Cortex* *, 49* (7), 1833-43.
26. Padovani A, B. B. (2006). Diffusion tensor imaging and voxel based morphometry study in early progressive supranuclear palsy. *J Neurol Neurosurg Psychiatry.* *, 77* (4), 457-63.
27. Pardini M, H. E. (2009). Olfactory function in corticobasal syndrome and frontotemporal dementia. *Arch Neurol.* *, 66* (1), 92-6.
28. Pereira JB, J. C.-R. (2009). Neuroanatomical substrate of visuospatial and visuoperceptual impairment in Parkinson's disease. *Mov Disord. , 24* (8), 1193-9.
29. Price S, P. D. (2004). Voxel-based morphometry detects patterns of atrophy that help differentiate progressive supranuclear palsy and Parkinson's disease. *Neuroimage* *, 23* (2), 663-9.
30. Ramírez-Ruiz B, M. M. (2007). Cerebral atrophy in Parkinson's disease patients with visual hallucinations. *Eur J Neurol.* *, 14* (7), 750-6.
31. Specht K, M. M.-H. (2005). Voxel-based analysis of multiple-system atrophy of cerebellar type: complementary results by combining voxel-based morphometry and voxel-based relaxometry. *Neuroimage* *, 25* (1), 287-93.
32. Summerfield C, J. C.-P.-A.-R. (2005). Structural brain changes in Parkinson disease with dementia: a voxel-based morphometry study. *Arch Neurol. , 62* (2), 281-5.
33. Tir M, D. C. (2009). Motor-related circuit dysfunction in MSA-P: Usefulness of combined whole-brain imaging analysis. *Mov Disord.* *, 24* (6), 863-70.
34. Tzarouchi LC, A. L. (2010). Voxel-based morphometry and Voxel-based relaxometry in parkinsonian variant of multiple system atrophy. *J Neuroimaging* *, 20* (3), 260-6.
35. Whitwell JL, D. J. (2013). Neuroimaging comparison of primary progressive apraxia of speech and progressive supranuclear palsy. *Eur J Neurol.* *, 20* (4), 629-37.
